# “The way I am treated is as if I am under my mother’s care”: qualitative study of patients’ experiences of receiving hospice care services in South Africa

**DOI:** 10.1186/s12904-020-00605-1

**Published:** 2020-07-01

**Authors:** Konstantina Vasileiou, Paula Smith, Ashraf Kagee

**Affiliations:** 1 Department of Psychology, University of Bath, 10 West Building, Bath, BA2 7AY UK; 2 Department of Social Work, University of West Attica, 12241 Athens, Greece; 3 Department of Psychology, Stellenbosch University, Private Bag X1, Matieland, Stellenbosch, 7602 South Africa

**Keywords:** Palliative care, Hospice, Patients, Cancer, South Africa, Interviews, Qualitative study

## Abstract

**Background:**

Research on the patient experience of receiving palliative care across a number of settings is increasing, but the majority of these investigations are situated within the context of developed countries. There is limited research from resource-limited countries, especially with regard to patients with cancer who receive hospice care. The present study explored the lived experience of attending hospice care facilities in South Africa to develop a bottom-up understanding from the perspectives of patients themselves.

**Methods:**

A qualitative cross-sectional study was designed to examine how patients experienced receiving hospice care We conducted in-depth, semi-structured interviews with thirteen, purposively selected patients living with terminal cancer and receiving in-patient or day care palliative services from a hospice organisation in South Africa. We used inductive thematic analysis to analyse the data.

**Results:**

We identified three themes that reflected a process of transformation that was experienced by participants during their engagement with the hospice services. The first theme describes participants’ initial reluctance to be linked to the hospice as a result of the stereotypic perceptions of hospice as being associated with death and dying. The second theme presents the perceived positive impact on patients’ physical and psychosocial wellbeing which resulted from the highly valued interactions with staff and other patients as well as patients’ engagement with creative activities. The final theme delineates the transformation of hospice into a second ‘family’ and ‘home’ and the restoration of an identity that expands beyond the ‘sick’ role.

**Conclusions:**

Receiving hospice care that sensitively attends to patients’ psychosocial and physical needs helps people to re-create a sense of homeliness within the world, re-orient themselves toward a meaningful life and re-configure their relationship with self. Patient experience of receiving hospice care in South Africa does not appear dissimilar to that reported by patients in resource-rich countries, suggesting underlying commonalities. There is a need for raising awareness and educating the public about what palliative care can offer to those in need. Public health campaigns could help reduce the stigma attached to palliative care, deflect negative perceptions, and communicate the benefits for patients, families and communities in culturally sensitive ways.

## Background

South Africa is among the few African countries that presents notable palliative care achievements. Integration of palliative care services with the mainstream healthcare provision has been attained to some degree [1, 2] with such services being offered in most hospitals throughout the country [2]. The operation of around 150 hospices [3] funded primarily by the charitable sector without income from government sources, is in place. Hospice care in South Africa is provided in three main ways [4]: (a) home-based care, (b) community centres and (c) in-patient units. Home-based care is provided by mobile teams of trained caregivers who visit people’s homes and educate families how to care for their patient who is typically homebound. Community centres are hospice bases within the community offering services to palliative care patients who are reasonably well and mobile. In-patient hospice units offer 24-h palliative care to patients whose pain is difficult to control, whose family needs respite, or patients who lack support systems at home. In-patient units have a limited number of beds and admit patients on specific criteria [4]. Hospices rely on community support for financial and human resources, including healthcare professionals’ capacity to volunteer [5]. Like other African countries [6], limited and/or unsustainable funding, opioids constraints and people’s perceptions of palliative care remain the key challenges for the field in South Africa [3, 5].

Whilst palliative care has mostly centred on addressing the needs from the HIV/AIDS epidemic, cancer is increasingly considered a public health concern, with 40,000 deaths yearly in South Africa [7]. Although there is a relatively developed, yet resource-constrained, health system in the country, low public cancer awareness and stigmatisation, as well as transport and financial challenges that obstruct patients’ timely access to diagnosis and curative treatment [8] make palliative care a vital alternative to alleviating suffering [9]. Yet, inadequate cancer pain relief is a significant problem in South Africa [10] even among patients receiving palliative care [11, 12]. Alongside pain, psychological distress is commonly reported [11], highlighting the need to understand patients’ psychological experiences of engaging with palliative care services from their perspective.

### Patient experience of receiving hospice care

Palliative care provision in the hospice setting can have several advantages for patients. In-patient hospice care during the last 6-months of life is associated with improved patient care experience, including satisfaction and pain control, and decreased mortality [13]. Patients value receiving suitable care whilst still enjoying the company of loved ones, but without placing a burden on them relating to their dying process [14]. They further appreciate the psychosocial climate and high-quality communication [15] whilst in-patient hospice care has been assessed more favourably by bereaved relatives compared to in-patient hospital care [16]. Positive patient experiences are further reported concerning hospice day care services [17, 18]. Patients report high levels of satisfaction with the care they receive and appreciate have their symptoms assessed when needed [17]. A person-centred approach which reduces isolation and feelings of loneliness, increases social support, encourages communication and provides activities are aspects that day care attendants mostly value [17, 18].

### The aim of the present study

Evidence about the patient experience of receiving hospice care is growing [13–15, 17, 18] but this research has almost exclusively been conducted in developed countries, raising questions as to what extent this knowledge might be applicable to resource-limited, and culturally different, national settings. Moreover, much of the literature on palliative care provision in Africa provides a top-down picture that addresses broad, policy-relevant questions [1–3, 6, 9, 19]. Whilst bottom-up research focusing on palliative care patients in South Africa is increasing, it focuses on pain control and management [10–12], quality of life [20], and information needs [21]. Building on existing evidence, we sought to explore the lived experience of receiving hospice care in South Africa with a view to developing a deeper understanding from the standpoint of patients living with terminal cancer.

## Methods

### Design and research setting

A qualitative, cross-sectional study was designed that used in-depth, semi-structured, individual interviews. The research took place at a non-profit hospice organization located in Cape Town, South Africa. The organization has a 10-bed, in-patient unit which can admit patients for up to 2 weeks – and multiple times if necessary – and community hospices which provide day centre facilities and home-based care and support for patients and their families. After hours and on weekends community services are covered through telephonic support. In total, over 700 patients are served every month according to the organization’s 2017/2018 patient statistics. Palliative care personnel include nurses, doctors, social workers, spiritual counsellors, and community health workers who are assisted by a network of volunteers drawn from the local communities. As of August 2018, there were 169 staff and approximately 600 volunteers. In line with palliative care principles, the hospice offers a range of services including nursing support, physical, emotional, psychosocial and spiritual care, and bereavement support for patients’ families. The hospice cares primarily for patients diagnosed with cancer.

### Recruitment, participants, and ethical considerations

Access to the organisation was initially sought by the third author (AK), and following discussion about mutual interests and research the current study was developed. The study was advertised by the staff in both the community and in-patient settings. Staff approached potential participants who were considered to be well enough to take part in the study and explained the study to them. Any interested participants were given written information in both Africans and/or English so they could consider taking part. Concerning the in-patient unit patients, whilst one person from those approached initially expressed an interest in taking part in the study, at the time of conducting the interviews he was not well enough to be able to participate. Regarding the day care patients, the researchers do not have information about whether any person from those approached by staff and given the study information pack refused to take part.

The study interview took place during the week of 9th - 13th October 2017. A purposive sampling was used for any participants who were able to participate during that time. Our inclusion criteria were (a) patients who were receiving hospice care services either in the in-patient unit or the day care centres and (b) who were well enough to take part in the study. Our exclusion criterion was patients who were not well enough from a health status perspective to participate. In total, 13 palliative care patients (7 women, 6 men) took part in interviews. Two were in-patient unit patients at the time of the interview and the remaining 11 were attending the day centres. All were receiving treatment for cancer within a palliative care context. Table 1 presents some basic demographic and life situation information about participants as this was revealed by themselves during the interviews. The research received ethical approval from Stellenbosch University (Ref SU-HSD-003055) and the Hospice Palliative Care Association South Africa (Ref 03/17). All participants gave written informed consent before taking part in the interview and were informed about their right to withdraw at any time without needing to give a reason and without any consequences on their care.

**Table 1:** Participants’ basic information

*Pseudonym assigned*	*Gender*	*Marital Status*	*Life situation*	*Attendance to hospice*	*Research site*
Paul	Man	Married	Lives with his wife; has two daughters living abroad and one son living in the country	About a month	Day care centre
Sarah	Woman	Married	Lives with her husband; has two sons who do not live with them. Daughter-in-law provides support	Less than a year	Day care centre
Amy	Woman	Widow	Lives with her son who is divorced.	22 years	Day care centre
			Receives help from her eldest daughter and best friend		
Alan	Man	Single	Lives with his mother, who is the main caregiver, and his brother; has a 16 years old son but does not seem to be in much contact with him	Has been admitted twice at the in-patient unit	In-patient unit
Eric	Man	Married	Lives with his wife; children and grandchildren are living close by	2 weeks	In-patient unit
Jane	Woman	Divorced	Lives on her own	About a year	Day care centre
Carol	Woman	Not referred to	Her family lives upcountry; she has friends only close by.	One year and a few months	Day care centre
Nicole	Woman	Married	Lives with her husband and child; receives regular help from her sister	More than a year	Day care centre
John	Man	Married	Lives with his wife; receives support from his two daughters	Around 2 years	Day care centre
Joanna	Woman	Not clear	Has children; not clear if she cohabits	3 years	Day care centre
Helen	Woman	Not clear	Her sister is the main informal caregiver who lives close by	5 years	Day care centre
Harry	Man	Married	Lives with his wife; his three children have left home	About 8 months	Day care centre
Michael	Man	Married	Lives with his wife and his son	4 years	Day care centre

### Data collection and analysis

The second (PS) and third author (AK), two experienced qualitative researchers with an educational background in nursing and psychology respectively, carried out one-to-one interviews with participants in October 2017. PS is British, and AK is South African and both are middle-aged. Interviews took place in a quiet location in the day care centre or in-patient unit at a time that was convenient to participants and lasted between 15 and 40 min (average length 28 min). Researchers wrote down notes about their impressions of the interview after or as soon as possible after each interview. The researchers did not know the participants prior to the interview; once they met the researchers introduced themselves, explained their role, reiterated information about the purpose of the study, their involvement with it as well as ethical considerations, and invited participants to ask any queries they might have had prior to the commencement of the interview. All interviews were audio-recorded and transcribed verbatim by transcribers employed by Stellenbosch University. Transcripts were not returned to participants for comments or corrections, but transcription was checked by the researchers against the audio recordings.

An interview protocol consisting of open-ended questions had been developed after discussions with and input from staff in the organisation to guide the conversations (supplementary file 1 provides the interview protocol for the interested reader). Questions explored participants’ experience of receiving care since being admitted to the hospice; how they feel and cope since being cared for by the hospice; whether they might have any concerns about the present or the future; and what their needs for help and support might be. Most of the interviews were conducted in English (*n* = 11). One interview was conducted in Afrikaans by the third author and was later translated into English and one interview was conducted in Xhosa with the presence of an interpreter.

Thematic analysis was adopted to analyse the data using the six-phase process suggested by Braun and Clarke [22]. Thematic analysis is a suitable analytic approach for identifying “patterns of meanings across a data set” in a systematic manner. The present analysis employed a bottom-up, data-driven approach, which sought to identify how participants themselves made sense of their experiences of receiving care at the hospice. The analytic process proceeded as follows: (1) initially researchers familiarised themselves with the data through the repeated reading of transcripts. A summary for each transcript was produced at this stage and initial thoughts about the data were noted. Then, (2) data relevant to participants’ experiences of hospice care were assigned to developing codes by KV assisted by computer software (NVivo 10). Next, (3) semantically related codes were collated, and (4) themes and subthemes were developed. A preliminary report was produced at this point by KV which (5) was discussed among the authors in order to revise and refine the developing themes (supplementary file 2 provides an example of theme development). After revising and refining the themes, (6) the researchers concluded to the final analytic report which is presented below. A previous draft of this paper was shared with the hospice with a view to receiving feedback from the organisation and disseminating, if possible, the findings to those participants whom the hospice might have judged to be well enough at the time of us communicating our findings. Although we did not receive any feedback from patients, the thoughts of the organisation on our results did not produce any changes on the manuscript. Illustrative extracts are used below to exemplify the analytic points that are being made. All participants’ names are pseudonyms.

## Results

The analysis below is structured around three themes, which uncovered a process of transformation experienced by participants through their engagement with the hospice. The first theme describes the stereotypic perceptions of hospice held by participants before receiving hospice care and their initial hesitance to be admitted to the hospice. The second theme presents the positive experiences of receiving hospice care and the perceived psychosocial benefits derived through participants’ interactions with staff, other patients and their engagement with activities. The third theme outlines how the hospice is transformed into a place that resembles ‘home’ and ‘family’ and how participants’ self-concept is again expanded beyond the ‘sick role’.

### Association of hospice with death and dying and the less fortunate

The strong association of hospice with death and dying in people’s perceptions was repeatedly expressed by several participants, bringing to the forefront and reflecting the prevalent stereotypic conceptions of hospice that are circulated in society. Participants spontaneously described their negative initial reactions when they were recommended to receive hospice services, such as reluctance, scepticism or even unwillingness and disquiet to be admitted at the hospice. Hospice was constructed as the ‘final destination’ for the very sick and frail patients who waited for imminent death and who might not have the chance to see their loved ones again. John, a married man who was attending the day care centre stated:

*After all I decided, OK. In the first place, I don't want to come to [name] Hospice, 'cause in my mind was when you go to [name] Hospice, is your last.. it's like a...your destination. OK, from here, you never see your family again. That was my thought.*

Not only did participants articulate the association of hospice with death and dying as their personal view alongside their concomitant hesitancy to receive hospice services but they were acutely aware of the socially shared nature of these stereotypes of hospice. Jane, a woman with breast cancer, said:

*And the people are even seeing the hospice, [name of hospice] hospice van in my road. Came and picked me up, and they always think, "Hospice. Ah, you gonna die." And then they see the two hospice cars there. The sister came with a car, and the social worker came with a car. So now it's two hospice [cars]. Now something's gonna happen there. Because that is people's mentality.*

Apart from the strong association with death and dying, hospice was also considered to be a place for the disadvantaged and less educated patients and for people who do not have family and friends to care for them. The view that hospices normally admit patients of lower socio-economic status was expressed by a patient with a higher socio-economic background. Paul, who was an educated person, found it necessary to manage the negative reactions of his social circle and defend his positive experience at hospice, suggesting an underlying stigmatisation of the hospice.

*"You … you educated person like you … What are you doing at the hospice?" (laughs). But, uh honestly, doctor, … I said, "Why not?" "Hospice is rendering a service. And it's not just for anybody. Uh, it could be for the lesser fortunate. But it could be for the highest educated patient. So what's your problem? I haven't got a problem."*

Despite these negative conceptualisations of hospice and initial hesitation to be linked to its services, participants described how their views radically changed after being admitted to the hospice and receiving support. As Amy, a widow who attended the day care centre, stated:

*So they [the doctors] couldn't do nothing more. And since then I'm with … with the hospice. At first, I didn't want to come. I said, "Just leave it", but I'm glad I did. Lots of support.*

Indeed, in an effort to normalise hospice and dispute the association with death and dying, the hospice was sometimes equated with a ‘normal hospital’, i.e. a typical medical hospital, where patients might die from illnesses other than cancer.

### Experiences of hospice services and perceived impact on patients

The experience of receiving care at hospice was described as enormously positive and beneficial across all participants. Feelings of happiness, joy and gratitude were very clearly articulated and some wished they could attend the day care centre every day. They were eager to attend the hospice every week and highlighted how each weekly visit helped them endure the rest of the week. Participants stressed that the existence of hospice was extremely meaningful and important to them and asserted that the hospice had improved their life considerably. Indeed, a few participants whilst engaging in counterfactual thinking stated that their lives would be poorer without the services of the hospice, which resulted in an amplification of sentiments of appreciation and gratitude. Indeed having experienced such a positive impact, participants were prepared to highly recommend the hospice to people who might need it. Carol and Michael characteristically said:

*That is how I feel. But I must say, "Thanks to the Lord for hospice." 'Cause if there weren't a hospice, I don't know what would have happened to all the cancer patients, because we do need that tender loving care. We do need it. Just to give us strength to go forward. (Carol)*

*But I’m thankful that it has saved my life. (Michael)*

This profoundly constructive experience with hospice was largely attributed to interactions with staff and volunteers and the high quality of care and support that patients reported. A plethora of characterisations were used to describe staff and volunteers. They were depicted as caring, loving, compassionate, dedicated and committed to their work, interested in patients’ wellbeing, available and responsive to their needs, helpful, encouraging, friendly, able to truly listen to patients’ problems and concerns without being judgmental, and reliable. Alan, who was receiving services at the in-patient unit, stated:

*And the social worker. They ask me various questions. Want to have a conversation with me about.. "Alan, how are you feeling today?" and "What have you been doing the weekend?" and "What has this been doing for you?" and "How's that thing working for you?" and they always listening to you, your problems. "How are you feeling?" "Are you worried about something?". Speaking to the social worker. If you have some … some other problems where … where the social worker can help you. She actually goes out her way to help you.*

Indeed, for a few participants the quality of care at hospice was so exceptional that they felt they were being “loved” by staff, an experience that was unprecedented for some. As Helen spontaneously commented “the way I am treated [here] is as if I am under my mother’s care”.

Although participants’ accounts mostly focused on the highly valued psychosocial support that they received at hospice, they also noted the significance of receiving practical help from staff. John, for example, was astonished by hospice staff’s immediate responsiveness and presence at a time of his medical emergency.

*Even the doctor. One or two months ago, I got … got sick at home. Two months ago. And … everything was falling 'part. My whole body. Diarrhoea was so much. Uh. I go to the toilet's only blood that's passing. Instead of the popo is blood. Took me to hospital, [name] Hospital. And I was there at about … less than ten minutes. She … the doctor from [name of hospice], she was there. I said, "Amazed!" Less than ten minutes on my arrival she was there. That nurse were there. The social worker were there.*

Interactions with other patients were also crucial as they seemed to provide opportunities for social contact and companionship, especially for those who were living alone. According to participants, these interactions cultivated a sense of social connectedness and for some alleviated feelings of loneliness. Moreover, the shared understanding among patients as a result of their illnesses allowed some participants to seek and provide informational and emotional support. A few, noting that other patients might be in a worse medical condition, gained a broader perspective about their own health status and this helped them to have courage. The use of humour among patients was also important as it improved their mood and uplifted their morale. The strength of bonds patients developed with each other was most apparent when someone died, with several participants expressing sadness during such times. Sarah, reflecting on why she feels better since attending the hospice services, referred to the friendships she had developed at the day care centre:

*I think it's 'cause when you lie, just in the bed, you feel so lonely. Because it's just me and my husband in the house. Uh, he's busy now. He is cleaning the house, cooking the food, and whatever. And, uh nobody to talk to. It's only me and my husband. And, uh so here now we have.. every Monday there's other friends. I mean, I ha-.. my other friends called me. Sms one another, and what. But.. then these are now my best friends … because I see them. I know I'm going to see them next week. To see them again.*

Several participants stressed the benefit of engaging with creative activities in the hospice. They noted how engaging with arts and crafts helped distract themselves from thinking about their illness, feel productive and creative, and retain a sense of purpose and achievement in their lives. Indeed, a few patients whilst talking about their illness highlighted how much they had missed working since they were diagnosed and had to leave employment, suggesting the importance of being energetic, prolific and creative in the context of managing a terminal illness which deprived people of energy, control and independence. Carol characteristically said:

*And I crocheted my own … own blanket. And I was so proud of it. Even though, in my state that I was, I could achieve something. Hospice is about achieving something. And not think about your illness. And then, one of the ladies asked me, because I like knitting, but … since my illness I never even picked up a knitting needle. And then I started knitting. And I've knitted baby clothing and I've knitted a … adult jersey.*

Receiving hospice care was perceived to have a significant impact on patients’ physical and psychological health. As Jane stated “It will make a big difference in your life, and in your family’s life, and in your people”. Several patients explained how their physical health had considerably improved since being linked to the hospice whilst in some instances this improvement was also noted by patients’ family. Joanna commented:

*They see me as better, because yesterday, yesterday—was it on Thursday? No, on Tuesday, my daughter was saying, “mom, I can see the difference even due to the fact that you look healthier than before.”*

A sense of psychological improvement as a result of receiving care and support at the hospice was also strongly expressed. Participants described how they felt a sense of freedom from their concerns and reassurance; that progressively they were better able to cope with their illness; that they felt encouraged and stronger to carry on with life and manage their condition; and that they were more hopeful and optimistic. Amy and Alan reported:

*Sometimes … there's times you feel a bit depressed. But coming here and, no, that is all gone. (Amy)*

*I got time to think about my situation and think what's happening and … and emotional, how … how I felt about it. I've been thinking about many things and eventually I thought I should be strong. I'm being helped here. I had some physio here and had encouragement here and they were kind with you - helpful, which made you feel quite free and … you feel in a quite safe environment and that's why I actually encourage myself. (Alan)*

### From death and sickness to home and personhood

Participants felt that they were so well cared for at hospice that eventually the place was transformed into a ‘home’ and a second family. A sense of embracement and warm welcoming was largely reported even from the first visits to the hospice. Whilst talking about the staff and volunteers, Jane stated:

*And the volunteers … must salute the volunteers. Because we, they will … let … make you feel like you are by a second home here. This is my second family. I got a first family by the house, but hospice is my second family.*

Apart from feelings of familiarity, closeness, and warmth that home typically signifies, the quality of care also made participants feel valued and important, providing in this way self-affirmation. Nicole stated:

*I feel … they make you feel as if you are the most important person in the world. I love coming here.*

Ultimately, retaining or regaining a sense of self that was not overwhelmed by the illness and an identity of being ‘sick’ was something patients attained through their involvement with the hospice alongside the support of family and friends. Engaging in pleasurable and productive activities functioned as a distraction from illness and made salient the fact that patients could still achieve things and enjoy life. This emphasis provided a sense of normality and continuity at an identity level and restored a disrupted personhood that was, even if momentarily, released from sickness. Carol commented:

*You just carry on, and forget about how you feel at that moment. And the minute you start getting busy, that pain or the thought of you being sick, you don't even feel sick. That's how good hospice makes you look at yourself. And feel about yourself.*

## Discussion

The aim of this study was to explore the experiences of patients with terminal cancer of receiving hospice in-patient or day care by a non-profit organization in South Africa. The findings illustrated a process of transformation occurring both with regard to the place and the patients’ sense of self. Whilst participants described initial reluctance to be linked to the hospice as a result of the narrow and stereotypic conception of hospice as being associated with death and dying, the perceived psychosocial benefits derived from the highly valued interactions with staff and other patients as well as patients’ engagement with creative activities transformed hospice into a second ‘family’ and ‘home’ and helped patients restore an identity that expanded beyond the ‘sick’ role.

Our findings echo prior research which suggests the strong stigma attached to palliative care [23] and an association of palliative care with diminished hope and choice, increased dependency [23, 24] and ultimately death [25]. The participants of this study, reflecting retrospectively on their reactions to the initial recommendation of hospice services, strongly articulated the stereotypic perception of hospice as a place of death and their concomitant fear and reluctance to be linked to its services.

Our findings further demonstrate that engagement with the hospice generated particularly positive experiences for the patients. This is in accordance with previous studies that consistently showed increased patient satisfaction and appreciation, especially with day care hospice services [17, 18]. The benefits from the involvement with the hospice were seen to accrue from three elements. The first concerned the interactions with staff who were perceived to provide high quality, person-centred care that was compassionate and responsive to patients’ needs. This supports earlier research showing that palliative care staff’s behaviours of presence, reassurance and respect for choice were considered important [26]. Participants in this study saw their interactions with staff to fully embody these qualities. Contact with other patients was the second element that contributed to patients’ positive experiences. In line with existing evidence [18, 27], these interactions provided social support (i.e. informational, emotional), companionship, and a sense of social connectedness and belonging that alleviated feelings of loneliness and isolation. Indeed, the importance, but also the challenges, of preserving social connectedness at the end of life have recently been underlined in literature [28]. Finally, in line with existing research [18, 27], patients’ engagement with activities (e.g. creative activities, knitting) was the third element that was narrated to produce psychosocial benefits such as increased confidence and self-esteem, a sense of achievement, purpose and usefulness, distraction from the illness, as well as a space for social contact.

Through experiences of genuine human connection with both staff and other patients, responsive, reliable and empathic care, and opportunities to facilitate patients’ striving for living a meaningful life amidst negotiating a terminal illness [29], the hospice was ultimately transformed from a place of fear into a ‘home’ and a ‘second family’. This feeling of homeliness and sense of sheltering that hospice created, also observed in previous research [30], provided a safe point of departure from which patients could become re-attuned with themselves, re-connect with facets of their self that went beyond the ‘sick’ role, and restore a sense of continuity and wholeness at an identity level [18, 29]. In other words, and as Moore et al. [30] insightfully noted in their own study, patients appeared to find a sense of home both within the hospice and in themselves.

Being diagnosed with a terminal illness and living with the prospect of imminent death presents an existential challenge that fundamentally disrupts people’s relationship with time [31], place [30] and biography [32]. Our findings indicate that providing hospice care that values and sensitively attends to patients’ psychosocial and physical needs helps people to reconfigure their relationship with time so that they can visualise a future, re-create a sense of homeliness within the world, and re-orient themselves toward a meaningful life [29].

### Strengths and limitations of the present study

Our findings should be considered within the context of the study limitations. First, we interviewed people at a particular moment in time. Longitudinal research is needed to follow up on how these experiences of receiving hospice care might change as a function of different illness stages and of a deteriorating physical state. Related to this, although we sought to recruit in-patient unit participants, only two patients were physically well enough and willing to participate within the time constraints of our data collection period. These articulated equally positive experiences with the hospice services. The majority of informants attended the day care facilities and had a better functional state which might have produced more enthusiastic narratives than those of poorly functioning patients very near to death. Indeed, research with in-patient hospice informants illustrates the tensions, frustrations and disruptions some patients might experience whilst transitioning to the hospice [33]. As a consequence, the present findings should be considered reflective of a relatively high functioning patient sample. Moreover, since the majority of interviews were conducted at the hospice facilities, the location might account to some extent for the high level of positivity we observed in patients’ narratives.

Further, it should be noted that the interviews were relatively short which might have influenced the richness of the data. The researchers deliberately tried to keep the interviews short for two reasons: regarding the in-patient unit participants, the researchers were cautious not to burden these patients due to their poor health status. Concerning the day care participants, the researchers were mindful to disturb these patients’ routine as less as possible and not take time away from their scheduled activities in the centre. Data collection ceased on pragmatic grounds which related to the practical and time constraints of our study rather than data saturation. Although we are confident about the trustworthiness of the themes that we are presenting, the relatively short duration of interviews and small sample size as well as the noteworthy absence of less positive experiences than those overwhelmingly expressed by participants make us cautious about whether saturation was reached. Despite these limitations, the present research offers useful insight into how receiving hospice care in South Africa improves psychosocial wellbeing in patients with terminal cancer, expanding in this way the evidential base of an area that is poorly researched in resource-limited contexts.

### Implications for policy and practice

Given the negative public perceptions of hospice and palliative care – which were also evident in this study – the need for raising awareness and educating the public about what hospice can offer to those in need is clearly evident at the policy level. For instance, public health campaigns could help reduce the stigma attached to palliative care, deflect negative perceptions, and communicate the advantages for patients, families and communities in culturally sensitive ways. This would help expand conceptions of hospice care and highlight the value of such care for people managing a terminal illness. Healthcare professionals who recommend hospice care to patients should also be aware of these perceptions and make every effort to tactfully communicate the benefits which patients and their families can derive from such care.

## Conclusions

According to the findings of the present study South African patients’ experiences of receiving hospice care do not appear dissimilar from those reported by hospice patients in resource-rich countries. This suggests underlying commonalities in patient experience and the fundamental psychosocial issues reported by them. Receiving hospice care that carefully considers patients’ psychosocial and physical needs assists people to live with their terminal illness with as good quality of life as possible until they die.

## Supplementary information

**Additional file 1: Supplementary file 1***.* Patient interview schedule. It provides the interview protocol the researchers used to conduct the interviews.

**Additional file 2: Supplementary file 2***:* Example of theme development. The development of the first theme is provided as an example of our analytic process.

## Data Availability

The datasets generated and/or analysed during the current study are not publicly available due to the sensitive nature of the data but are available from the corresponding author on reasonable request.
