# Supplementary material for: “The way I am treated is as if I am under my mother’s care”: qualitative study of patients’ experiences of receiving hospice care services in South Africa
Source: BMC Palliat Care. 2020 Jul 1;19:95. doi: 10.1186/s12904-020-00605-1 (PMC7330936; doi:10.1186/s12904-020-00605-1)
Supplement: Supplementary file 1 — Additional file 1: Supplementary file 1. Patient interview schedule. It provides the interview protocol the researchers used to conduct the interviews. [file 12904_2020_605_MOESM1_ESM.docx]

# **Interview schedule**

First of all, I would like to thank you for taking the time to talk to me. As you know we are interested in understanding **your** experience since you have been cared for by the [name of the organisation] Hospice. I have here some topics which might be useful to discuss, but most important are your experiences.

I am going to record the interview so that I can listen to what you have said at a later date. However, if you want to stop the interview or the tape recorder at any time you can. All the information you provide will be strictly confidential. Your name will not be mentioned in any reports arising from this study.

## **Section One – Your experience of receiving care since you have been referred to the hospice**

1. So we can understand your experience of the care that you have had since you have been referred to [name of hospice], can you tell me a little about what has happened so far?

*Prompt:* for example, how long is it since you were admitted to [name of hospice]? How has this affected your own situation? How has [name of hospice] helped you?

2. Do you have any particular difficulties at the moment?

*Prompt:* Can you do most things for yourself or do you need help with some things such as dressing, eating? Do you have any physical difficulties? Do you have any emotional difficulties?

3. Do you feel you have enough information about what is happening at the moment?

*Prompt:* Have you been able to talk to enough doctors, nurses and other health and social care professionals about what is happening for you?

4. Have there been any changes in your relationship with the people who have cared for you at home since you have received help from [name of hospice]?

*Prompt:* Has there been any changes in your roles and responsibilities? Have you found it easier or more difficult to talk to each other about what is happening/everyday issues and decisions/your future?

## **Section Two – Your mood and how you are coping since you have been cared for by [name of hospice].**

5. Can you tell me a bit about how your mood has been since you have been cared for by [name of hospice]?

*Prompt:* Have you been more happy/sad/frustrated than usual? Have your friends/family commented on your mood? Have you experienced any increase in minor illness such as colds? Have you noticed any increased emotional responses – sense of tearfulness/lack of ability to laugh/sense of powerlessness?

6. How do you feel you are coping at the moment?

*Prompt:* Have you made any changes to your routine or activities? Have you tried to find out more information about your care or prognosis – if so how? Have you spent more time relaxing – if so how? Have you avoided thinking about the future or what might happen? Have you asked more people for help with things like shopping and gardening than you would normally?

7. Have you had someone who you can talk to about your concerns and emotions?

*Prompt:* if yes who, if no do you feel you would have liked to have someone to talk to? Was it helpful to talk about your concerns and emotions – e.g., did you find some new ways of coping/dealing with things?

## **Section Three – Current and future concerns**

8. Do you have any particular concerns about the present or the future?

*Prompt:* any medical concerns/emotional or social spiritual/ concerns/practical issues/financial concerns/worries about work/need for more information?

## **Section Four – Your need for help/support**

9. Have you managed to get the help or support you wanted?

*Prompt:* practical help/support – e.g., help with shopping/visiting the hospice/doing the gardening? Informational support – about your condition? Emotional support – someone to talk to/encourage you?

10. Has anyone been particularly helpful and supportive for you at this time?

*Prompt:* family and friends/staff at the hospice/work colleagues? What did they do that was especially helpful for you?

11. Can you tell me who is most important in helping you at the moment?

*Prompt:* family, friends, hospice staff, social care staff.

12. Finally do you have anything to add that would help us to understand your experience so far and the type of support that you would have found helpful?

**Thank you for your participation in this study.**
