# Supplementary material for: “The way I am treated is as if I am under my mother’s care”: qualitative study of patients’ experiences of receiving hospice care services in South Africa
Source: BMC Palliat Care. 2020 Jul 1;19:95. doi: 10.1186/s12904-020-00605-1 (PMC7330936; doi:10.1186/s12904-020-00605-1)
Supplement: Supplementary file 2 — Additional file 2: Supplementary file 2: Example of theme development. The development of the first theme is provided as an example of our analytic process. [file 12904_2020_605_MOESM2_ESM.docx]

## **Example of theme development**

| **Codes** | **Example of extracts** | **Theme** |
| --- | --- | --- |
| Negative reactions to the initial recommendation of the hospice services | *Participant: That was when he told me that I got six months and that, my…my, uh…a friend of us, a friend of us, told my husband, uh that I must now go to hospice.*  *Interviewer: Right, ok.*  *Participant: So hospice for me was now, "Oh! (disgust) I don't want to be…(laughs) think of a hospice. I don't want to be there." I was really upset, but I didn't say anything.* | Association of hospice with death and dying and the less fortunate |
| Association of hospice with death and dying | *And the doctor mentioned hospice. I was thinking of this. No man. If you go to hospice you gonna die there. Hospice is only for people who go lay there in the bed. So I said, "No." I said, "No, I'm not going to hospice." I said, "No, I'm not going to hospice because hospice is not for me." Hospice is for cancer patients that are like very, very sick. That was my…my version of hospice.*  *And then I said to her no, you know what…I don't think I want to go to hospice. Because the people who go to hospice, they die.*  *I didn’t want to come here, because I was saying it’s a place where you wait for the death.* |  |
| Association of hospice with the disadvantaged | *Participant: And everybody knows, in our street, that, uh... I'm...It's not a question of the high and mighty never gone...for to sink to this level, to...to be with the hospice…because…you don't sort of find the professional people here, if you know what I mean.*  *Interviewer: Right.*  *P: I don't know if they look upon it as a stigma. Uh, it's just a hospice (disdain in voice). And things like that. But to me, it couldn't have been better.*  *Interviewer: Right, ok.*  *Participant: Being part of the…being part of this set up.* |  |
| Association of hospice with people who do not have family | *Because I always think hospice is for people with cancer who don't have family at home.* |  |
| Change of negative perceptions of hospice after receiving the services | *Participant: I think people, if you explain to them they get a better thinking of hospice because now you think hospice is for people who go lay, because…people will lay there in [name of place]. When their time comes, when you can do nothing for yourself, then you go lay there.*  *Interviewer: Right.*  *Participant: So a lot of people think… like I also did think, hospice is a place…but it's not like that. It's…I got a different version now of hospice.* |  |
| ‘Normalisation’ of hospice | *Interviewer: So if somebody asked you now, then, how would you...what would you tell them the hospice is?*  *Participant: I will tell them, now...You...you don't...you don't die, if they tell you to go...maybe you won't die from cancer. You will die maybe from something else. While we belong here at hospice.*  *Interviewer: Right.*  *Participant: You see. So I will tell them, "No. There's…it is…it's just like a normal hospital. like…You go in for, you have high blood pressure, diabetes or whatever. It is normal.* |  |
